# Polyphenols and DNA Damage: A Mixed Blessing

**DOI:** 10.3390/nu8120785

**Published:** 2016-12-03

**Authors:** Amaya Azqueta, Andrew Collins

**Affiliations:** 1 Department of Pharmacology and Toxicology, Faculty of Pharmacy, University of Navarra, C/Irunlarrea 1, 31009 Pamplona, Spain; 2 IdiSNA, Navarra Institute for Health Research; 3 Department of Nutrition, Institute of Basic Medical Sciences, University of Oslo, PB 1046 Blindern, 0316 Oslo, Norway; a.r.collins@medisin.uio.no

**Keywords:** polyphenols, flavonoids, human studies, in vitro, in vivo, DNA damage, DNA protection

## Abstract

Polyphenols are a very broad group of chemicals, widely distributed in plant foods, and endowed with antioxidant activity by virtue of their numerous phenol groups. They are widely studied as putative cancer-protective agents, potentially contributing to the cancer preventive properties of fruits and vegetables. We review recent publications relating to human trials, animal experiments and cell culture, grouping them according to whether polyphenols are investigated in whole foods and drinks, in plant extracts, or as individual compounds. A variety of assays are in use to study genetic damage endpoints. Human trials, of which there are rather few, tend to show decreases in endogenous DNA damage and protection against DNA damage induced ex vivo in blood cells. Most animal experiments have investigated the effects of polyphenols (often at high doses) in combination with known DNA-damaging agents, and generally they show protection. High concentrations can themselves induce DNA damage, as demonstrated in numerous cell culture experiments; low concentrations, on the other hand, tend to decrease DNA damage.

## 1. Introduction

For many years now it has been recognised that fruits and vegetables play an important role in preventing or alleviating the effects of various chronic diseases, notably cardiovascular disease and various cancers. The mechanism(s) of this protection is still not clear. A common explanation is the so-called antioxidant hypothesis; oxidative stress is a factor in many diseases; fruits and vegetables contain various phytochemicals with antioxidant properties, and so these are likely to be the agents of protection. This is clearly a simplistic hypothesis; phytochemicals have been shown to have a wide array of influences on the physiological processes of human cells, and reducing them to sources of antioxidant activity is misguided and misleading. A meta-analysis of clinical trials indicates that antioxidant phytochemicals taken as supplements have no beneficial effect on mortality and may even increase it [1]. In natural plant foods, of course, phytochemicals of different kinds are present, acting in concert, often in all likelihood synergistically, and so studies of whole foods or extracts are particularly valuable. The reductionist approach (looking at individual components) is still popular, however, as evidenced by the large number of studies of individual phytochemicals, and by the growing catalogue of plant species that have been extracted and tested for potential health-promoting effects using a range of molecular markers. DNA damage is one of the most commonly employed such markers, in the reasonable belief that a decrease in DNA damage—as the initiating event of carcinogenesis—must signify a decrease in cancer risk.

Currently, the most popular assay for DNA damage at the cellular level is single cell gel electrophoresis, or the comet assay [2]. It is based on the ability of a strand break (SB) to relax supercoiling in a loop of DNA, thus allowing the DNA to extend to the anode during electrophoresis forming a comet-like image in which the relative intensity of the comet tail reflects the break frequency. Strand breakage is a feature of some but not all kinds of DNA-damaging agent. Reactive oxygen species, in particular, tend to cause damage to DNA bases. An example of base oxidation is 8-oxo–7,8-dihydroguanine (8-OH–Gua). This is converted to a SB by the action of formamidopyrimidine DNA glycosylase (Fpg)—a bacterial repair enzyme, and a simple modification of the comet assay, incorporating an enzymic digestion of the DNA after lysis of cells in agarose—allows the detection of oxidised purines. An analogous enzyme, endonuclease III (or Nth) converts oxidised pyrimidines to SBs. In the search for antioxidant protection of cells against such damage, it is surprising that so few published studies actually use the enzyme-modified comet assay.

The measurement of resistance to H_2_O_2_-induced damage is a good marker of cellular antioxidant status. Typically, cells are exposed in vitro to 50–100 μM H_2_O_2_ for a brief period, and the yield of SBs is measured with the basic comet assay; the lower the break frequency, the higher the antioxidant status.

The base 8-OH–Gua and the nucleosides 8-OH–Guo and 8-OH–dGuo can be detected in tissues, but are more commonly measured in urine, plasma or serum, using high performance liquid chromatography (often linked with mass spectrometry) and antibody-based techniques (ELISA or immunohistochemistry). In the tables and text that follow, we use the abbreviation 8-OH–G to cover all three compounds, as the oxidised base is the common factor. They are markers of oxidative stress [3,4]; free 8-OH–Gua can arise through cellular DNA base excision repair, though the origin of the oxidised nucleosides is not certain.

γ-H2AX is the phosphorylated form of histone H2AX, which appears at the site of DNA damage (particularly double SBs); it is detected by immunocytochemistry [5], or sometimes by immunofluorescence combined with flow cytometry [6], and is a sensitive damage indicator.

Unrepaired DNA damage can result in alterations at the level of chromosomes. Classically, chromosome aberrations (chrom abs) were studied as an index of genomic instability, but now the presence of micronuclei (MN: fragments of chromosomes or whole chromosomes that segregate as discrete bodies at mitosis) is a more common marker [7]. Both chrom abs and MN have been confirmed—in long-term human clinical studies—as prospective markers of cancer risk [8,9].

Here, we summarise the results of recent investigations of effects of polyphenols—a very broad class of phytochemicals—on DNA damage, at the level of humans, in animal experiments, and in in vitro studies using cultured (usually human) cells. 

## 2. Methods

In this review, we have concentrated on papers published from 2010 to the present. We used PubMed with the followings terms in the title or abstract: polyphenols/polyphenol/flavonoids/flavonoid combined with DNA damage/DNA protection/DNA repair. We found a total of 386 papers. We have concentrated on papers where the effect of polyphenols, in the form of real food, plant extract or pure compound, is tested in cell culture, animals and humans. We have excluded papers where only gene expression was studied, papers specifically focused on other diseases than cancer, and papers, for example, with deficient experimental design. Papers in which the main interest is in the induction of apoptosis were also excluded.

The reports are summarised in tables according to whether they deal with whole foods (or drinks) (Table 1), with extracts of plants (Table 2), or with single phytochemicals (Table 3). Studies are further classified as ‘in humans’, ‘in vivo’ (animal studies), or ‘in vitro’ (experiments with cultured cells). Extracts and phytochemicals are, where possible, grouped according to functional, chemical or botanical relationships (such as ‘tea and coffee related compounds’, or ‘flavonoids’, or ‘*Lamiaceae*’). We have generally excluded in vitro experiments with plants or compounds appearing in just one or two publications, unless they fall into one of these groups.

## 3. Results

### 3.1. Whole Foods and Drinks

Relatively few investigations of effects of whole foods on genetic damage endpoints have been published. A variety of fruit-derived drinks as well as tea (though this could be considered an extract), and dark chocolate, were tested in human supplementation trials. A decrease in urinary 8-OH–G was seen in overweight or obese adults supplemented with orange juice [10] but levels of plasma 8-OH–G in triathletes were too low to see any effect of Aronia-citrus juice [11]. De-alcoholised wine given daily for one month was without effect on DNA SBs or Fpg-sites in peripheral blood mononuclear (PBMN) cells of post-menopausal women [13]. However, a daily blueberry drink taken for 6 weeks protected PBMN cells from H_2_O_2_-induced damage, but had no effect on SBs or DNA repair capacity [14]. Malhomme de la Roche et al. [15] found that ingestion of green tea protected PBMN cells challenged ex vivo with UV(A)/VIS (ultraviolet(A)/visible) radiation, but only in some subjects, described as responders. Alleva et al. [16] gave a honey supplement to humans exposed to pesticides, and found, after two weeks’ supplementation, lower levels of EndoIII- and Fpg-sensitive sites in lymphocytes as well as an enhanced capacity for DNA repair. Dark chocolate induced a transient protection against H_2_O_2_-induced DNA damage in PBMN cells ex vivo [12].

Most of the animal studies have looked at the possible protection afforded by polyphenol-rich foods or drinks against DNA damage induced by treating the animals (rats or mice) with known carcinogens such as doxorubicin (Dox), n-nitrosodiethylamine, or sodium arsenite. Protection was claimed with *Chrysobalanus icaco* fruit [17], Piquia pulp [19], Açai pulp [20], and tea [18,22]; but cloudy apple juice actually increased SBs and had no effect on nitrosamine-induced damage [21]. Treatment of hyperlipidemic rats with spinach increased the resistance of blood cells ex vivo to H_2_O_2_-induced damage [23].

Experiments with cultured cells and whole foods/drinks are understandably rarely performed. Incubation of PBMN cells with green tea decreased DNA damage at low concentrations but increased it at the highest concentration tested (representing 71 mM catechins) [24]. Various honeys afforded slight protection of HepG2 cells against SBs produced by treatment with certain organic carcinogens [26]. A Chinese herbal preparation caused SBs in mouse lymphoma cells and rat fibroblasts, but at extreme concentrations (1–13 mg/mL) [25]. 

### 3.2. Extracts of Plants

#### 3.2.1. Tea-Related Extracts

One human trial and several animal experiments have been reported with tea-related extracts. Post-menopausal women with osteoporosis were supplemented with green tea polyphenols for 6 months; the level of urinary 8-OH–G decreased [27]. Xu et al. [28] found a decrease in 8-OH–G in rats given a very high dose of green tea polyphenols. Protective effects of green tea extracts against genetic damage were reported by Garcia-Rodriguez et al. [30] in mice treated with Cr(IV); and by Pu et al. [31] in rats treated with acrylonitrile. Katiyar et al. [29] found that green tea polyphenols promoted the repair of UV-induced DNA lesions in mice proficient in nucleotide excision repair (NER), but not in NER- mice. Two studies with cultured cells have found increases in DNA SBs induced by green tea extract; Prasad et al. [35] in melanoma cell lines (though at rather high concentrations), and Durgo et al. [36] in a human laryngeal carcinoma cell line.

#### 3.2.2. Lamiaceae Family Plants

The *Lamiaceae* family includes many plants used as culinary herbs, and so they have been grouped together here. All publications in our search deal with effects in cell culture.

Calo et al. [39] tested an extract of *Thymus vulgaris* (and thymol in parallel) on keratinocytes irradiated with UV(A) or UV(B); they found a decrease in SBs, though no effect on MN or γ-H2AX foci. A similar protective effect was reported by Cornaghi et al. [38] in a human skin model exposed to UV(B). A citrus and rosemary extract (but at high concentrations) decreased the frequency of MN induced by X-rays in human lymphocytes, and decreased UV(B)-induced SBs in keratinocytes [37]. This last group also tested lemon balm extract on UV(B)-irradiated keratinocytes and found a decrease in SBs (at a high concentration) and in γ-H2AX foci at a more moderate concentration [40]. Thirugnanasampandan et al. [42] studied three *Lamiaceae* species; HepG2 cells were incubated for 4 h with an extract before treating with CdCl_2_. Dose-dependent decreases in SBs were seen with all three (though even the lowest concentration tested was high). An extract of *Ocimum sanctum* (a form of basil) was tested by Venuprasad et al. [41] on human neuroblastoma cells; it protected against H_2_O_2_-induced SBs (at a high concentration).

#### 3.2.3. Honey-Related Extracts

In parallel experiments to their human honey trial, Alleva et al. [16] showed that pre-treatment of cells with honey extract protected against pesticide-induced DNA damage and inhibition of DNA repair. Propolis extract (at high concentration) decreased the frequency of γ-ray-induced SBs in fibroblasts [50], and yet—at a much lower concentration—it caused oxidative damage (SBs measured with Fpg and EndoIII together in the comet assay) in a human cancer cell line, which was suppressed by antioxidants or catalase and so was imputed to the production of H_2_O_2_ [51]. 

#### 3.2.4. Fruits and Berries

All papers on extracts of fruits and berries reviewed here describe cell culture experiments and with one exception they have made use of high to extremely high extract concentrations. The extract of one Australian fruit (among several studied) caused an increase in MN [48]. Other reports are of protection against oxidation damage caused by H_2_O_2_ [43,44,46]; or tert-butyl-hydroperoxide (t-BOOH) [45,49]. The exception to usage of high doses is a report by Bellion et al. [47] with apple polyphenol extracts; they found that 24 h pre-incubation of Caco2 cells decreased the DNA damage induced by menadione (low concentrations actually giving the greatest protection). 

#### 3.2.5. Miscellaneous Plant Extracts

Animal experiments with various plant extracts have shown protection against SB production in liver cells of pyrogallol-treated rats (at very high doses of extract) [34]; accelerated rejoining of γ-ray-induced DNA SBs [33]; and a decrease in pyrimidine dimers in the skin of UV(B)-irradiated mice [32].

## 4. Isolated Phytochemicals

### 4.1. Compounds Related to Tea and Coffee

Compounds tested—caffeic acid, chafuroside B, chlorogenic acid, ellagic acid, epicatechin, epicatechin gallate, epigallocatechin gallate, theaflavin.

One human trial with epigallocatechin gallate in prostate cancer patients showed no significant effect on 8-OH–G in leukocytes [52]. Animal studies with single polyphenols have generally involved treating mice or rats with a known DNA-damaging agent and looking for protection against DNA breaks, MN and chrom abs. Generally, protection is seen [54,57] though in some cases at rather high doses [56,63]. Pretreatment of rats with epicatechin reduced the level of DNA breaks induced in bone marrow cells by the topoisomerase poison etoposide [55]. High concentrations have also been used in in vitro experiments with cultured cells, and have given increases in SBs and γ-H2AX foci [71] and in γ-H2AX and 8-OH–G [58]. Kumar et al. [79] found that a combination of ellagic acid with curcumin (25 μM each) caused SBs while the separate compounds had no significant effect. A decrease in (background) SBs with epigallocatechin gallate or epicatechin gallate was reported by Durgo et al. [36] at 48 but not 72 h. At more reasonable concentrations, the results are mixed: decreases in UV(B)-induced SBs [70] and cyclobutane pyrimidine dimers [72]; decreases in H_2_O_2_-induced SBs and MN [74]; a decrease in SBs induced by cumene hydroperoxide [75]; but SBs and Fpg-sites increased in tetradecanoyl phorbol acetate (TPA)-stimulated neutrophils [78] and an increase in SBs with ellagic acid in prostate cancer cells was reported by Vanella et al. [73] at concentrations of 9 μM in one of the cell lines but higher concentrations in two other lines.

### 4.2. Curcumin

Curcumin was examined alongside epicatechin by Papiez [55]; at high concentration (up to 0.2 g/kg/day), it decreased DNA damage in the bone marrow of rats treated with etoposide. In cultured cells, curcumin at rather high concentrations caused SBs [85,86] and γ-H2AX foci [83]. The production of SBs in combination with ellagic acid was noted above [79]. Lewinska et al. [81] reported a pro-oxidant effect of curcumin at concentrations of 10 μM (and below), indicated by an increase in 8-oxo–G in smooth muscle cells. Sebastia et al. [80] compared effects of curcumin on human lymphocytes, both stimulated by TPA and unstimulated, and γ-irradiated. In non-cycling cells, the phytochemical was radioprotective (decreasing the level of premature chromosome condensation), whereas in cycling cells it acted as a radiosensitiser, increasing the frequency of chrom abs.

### 4.3. Resveratrol

At high concentration, in human lymphocytes, resveratrol decreased the frequency of chromosome aberrations caused by aflatoxin [87]. At a lower concentration, it protected human epithelial cells against SBs and MN induced by sodium arsenite [88], and rat astrocytes against SBs caused by ethanol [91]. However, a low dose enhanced the frequency of γ-H2AX foci after ionising irradiation of prostate epithelial cells [90]. A moderately high concentration applied to colon cancer cells caused γ-H2AX foci, apparently as a result of topoisomerase II poisoning [89]. SBs as well as Fpg-sites were increased in non-cycling cells but decreased in TPA-stimulated, cycling cells [78]. In contrast, Sebastia et al. [80] found that, as with curcumin, effects of resveratrol on irradiated lymphocytes differed depending on whether the cells were non-cycling (showing a decrease in premature chromosome condensation), or cycling (in which it had the opposite effect, acting as a radiosensitiser, increasing chromosome aberrations).

### 4.4. Flavonoids

Kozics et al. [97] performed a useful comparative study of 10 flavonoids, concluding that their effectiveness at protecting against B(a)P-induced SBs and MN depended on their chemical structure. Tested over a relatively low concentration range, fisetin, quercetin, galangin, kaempferol and luteolin (in order of decreasing effectiveness) were more effective than chrysin,7-hydroxyflavone, 7,8-dihydroxyflavone or baicalein, while rutin was without effect.

Among the flavonoids, quercetin appears most often in this survey. At low concentrations, SBs induced by aflatoxin B1 (AFB1), methyl methanesulphonate (MMS), Dox, HgCl_2_ or methyl mercury in HepG2 cells were decreased [100,101]. At high concentrations, quercetin and also rutin (glycoside of quercetin with rutinose) caused massive γ-H2AX foci, probably reflecting lethality [94], and yet they decreased DNA damage (SBs) induced by food mutagens PhIP and IQ [96].

Quercitrin, the rhamnose glycoside of quercetin, protected mouse epidermal cells against UV(B)-induced SBs [102]. Rutin at low concentrations showed the same protective effect as quercetin on HepG2 cells treated with AFB1, MMS or Dox [100]; at much higher concentrations, it caused SBs, but still protected against MN induced by B(a)P [93].

The myricetin rhamnoside, myricitrin, at high concentrations, induced MN in TK6 cells; the aglycone myricetin, being more cytotoxic, was tested at lower concentrations, and gave equivocal results [65]. Kaempferol at high concentrations induced SBs [95,99], as did fisetin [98]. Galangin and chrysin caused base oxidation at the moderate concentration of 20 μM [51], while a low concentration of naringin was protective against cadmium-induced chromosome aberrations [92]. Apigenin at a high concentration decreased SBs, chrom abs and MN [69].

## 5. Discussion and Conclusions

Many of the papers that we have reviewed report experiments with high or very high concentrations of phytochemicals. When investigating the role of phytochemicals in normal human nutrition, the aim should always be to study concentrations close to those likely to be present in humans as a result of dietary intake. As a rule of thumb, we have assumed this concentration to be in the low micromolar range. Many papers quote concentrations in μg/mL. To convert these concentrations to micromolar, again as a rule of thumb, we have assumed a molecular weight of 500; then 1 μg/mL = 2 μM. We would regard a concentration of over 20 μM or 10 μg/mL as high, and over 50 μM or 25 μg/mL as very high. Clearly, in functional foods or phytochemical supplements, the concentration is likely to be higher than in natural foods, and experiments showing genotoxicity of phytochemicals at high doses should at least serve as a warning to designers of functional foods.

It is always instructive to carry out experiments over a range of concentrations. Often, in the case of micronutrients in general, the dose–response curve is U-shaped, i.e., a beneficial effect at low concentrations changes to a detrimental effect at higher concentrations, and this tendency is clear in many of the reports described here.

Of course, if genotoxicity is specifically directed to cancer cells while healthy cells are unaffected, it is regarded as beneficial, and it is evidently the aim of some of the papers that we have reviewed to identify plant extracts or particular polyphenols that have such targeted action and so might have potential value as therapeutic agents. The differential response of cycling vs non-cycling cells to certain polyphenols might be exploited therapeutically in targeting dividing cancer cells. 

With such a wide-ranging set of phytochemicals, not to mention the variety of test systems, experimental designs and assays applied in their study, it is difficult to generalise. However, high concentrations are likely to show DNA-damaging effects, while also in many cases protecting cells against damaging effects of other agents, apparently acting as pro-oxidants when present alone, but as anti-oxidants in combination. This is not a novel observation: many years ago, Duthie et al. reported DNA-damaging effects of quercetin at 50 μM [103] alongside an ability to protect cells against H_2_O_2_-induced DNA damage at concentrations of 10–50 μM [104]. Low concentrations are generally protective, in some cases even decreasing the already low background level of cellular DNA damage.

To summarise, results reported in the recent literature, on the whole, lend support to the hypothesis that dietary polyphenols protect the body against the effects of reactive oxygen species on DNA integrity, but do so reliably only when present at low concentrations. We recommend that greater attention be paid to the concentrations used, particularly in in vitro experiments, if the results are to be extrapolated to issues of human health. An important consideration when extrapolating is that plant foods contain a variety of micronutrients which might be expected to act in concert, whereas most experiments are carried out with single compounds. In this respect, there are clear advantages in using plant extracts or whole foods, though this approach does present practical difficulties. We also recommend that, since oxidative damage to DNA, and its prevention, are of major concern, the modified comet assay incorporating Fpg or EndoIII should be employed, since it provides increased sensitivity and specificity.

## Figures and Tables

**Table 1:** Effects of whole foods or drinks on various genetic damage endpoints, in humans, in animals (‘in vivo’), and in cultured cells (‘in vitro’).

Reference	Material Tested	Analysis	Assays	System	Concentration/Dose	Result
**In Humans**						
[10]	Orange juice	Polyphenols	8-OH–G in urine by ELISA	Overweight/obese humans	300 or 745 mg/day (12 weeks)	8-OH–G ↓
[11]	Aronia-citrus juice	Flavonones, flavones, antocyanins etc.	8-OH–G in plasma by UHPLC-MS/MS	Triathletes (supplemented and placebo groups)	200 mL/day (45 days)	Inconclusive—levels of DNA damage products too low
[12]	Dark chocolate	Polyphenols	Comet assay	Healthy subjects: PBMN cells	860 mg/day (2 weeks)	H_2_O_2_-induced SBs ↓ (short-term—2 h—only)
[13]	De-alcoholised wine	Anthocyanins, flavonols etc.	Comet assay with Fpg	Post-menopausal women; peripheral blood lymphocytes	500 mL/day (1 month)	No effect
[14]	Wild blueberry drink	Phenolic acids and anthocyanins	Comet assay + Fpg; H_2_O_2_resistance (comet assay); DNA repair (in vitro comet assay)	Subjects with cardiovascular risk factors: PBMN cells	375 mg anthocyanins/day (6 weeks)	No effect on DNA SBs. Fpg-sensitive sites ↓; H_2_O_2_ resistance ↑; no effect on repair
[15]	Green tea		Comet assay	Healthy subjects: PBMN cells 30, 60, 90 min after ingestion, exposed ex vivo to UV(A)/VIS radiation	Single 540 mL dose	Protection against UV(A)/VIS-induced DNA SBs seen in ‘responders’
[16]	Honey	Phenolic compounds	Comet assay with EndoIII, Fpg	Pesticide-exposed humans	2-week honey supplementation (50 g/day)	DNA repair ↑, EndoIII and Fpg sites ↓
**In Vivo**						
[17]	*Chrysobalanus icaco* fruit	Polyphenols, Mg, Se	Comet assay on blood and MN assay on bone marrow and PBMN	Rats + Dox	Up to 0.4 g/kg/day for 14 days	Blood cells; DNA SBs ↓. Bone marrow, blood cells; MN ↓
[18]	Green and black teas		8-OH–G on liver by HPLC	Swiss albino mice + Na arsenite	2.5% of 0.5 g dry leaves/5 mL of boiled water (equivalent to human consumption of 1 cup). 22 days.	Protection (8-OH–G ↓)
[19]	Piquia pulp	Phenolic compounds, carotenoids	Comet assay on liver, kidney, heart cells MN on bone marrow and PBMN cells	Rats + Dox	75, 150, 300 mg/kg/day for 14 days	Protection against DNA SBs and MN formation: lowest dose tends to be most effective
[20]	Açai pulp	Phenolic compounds, carotenoids	Comet assay on liver, kidney and PBMN cells: MN on bone marrow and PBMN cells	Mice + Dox	3.33,10, 16.7 g/kg/day for 1 or 14 days	Protection against DNA SBs and MN formation: 14 days pretreatment more effective
[21]	Cloudy apple juice	Polyphenols	Comet assay on liver cells	Rats	10 mL/kg/day for 28 days	DNA SBs ↑ and no effect on N-nitrosodiethylamine-induced damage
[22]	Green tea	--	Comet assay on intestinal cells	Rats + As	10 mg/mL in water for 28 days	Claim protection
[23]	Spinach	Total polyphenols	Comet assay on leukocytes	Hyperlipidemic rats	5% (powder) in diet, for 6 weeks	H_2_O_2_-induced DNA SBs in leukocytes ↓
**In Vitro**						
[24]	Green tea	--	Comet assay with Fpg	Human PBMN cells	7–71 µM catechins	DNA damage ↓ at lower concentrations but ↑ at highest concentration
[25]	Herbal preparation	Total phenolics	Comet assay	YAC-1 (mouse lymphoma) cells	1–13 mg/mL	DNA SBs ↑ at 8.7 mg/mL
				Rat fibroblasts	1–13 mg/mL	DNA SBs ↑ at 2.2 mg/mL
[26]	Various honeys	--	Comet assay	HepG2 (human liver carcinoma) cells treated with B(a)P, PhIP, nitrosamines	0.1–100 mg/mL	Slight decreases in DNA SBs in most cases, not dose-dependent

PBMN: peripheral blood mononuclear; SB: strand break; Fpg: formamidopyrimidine DNA glycosylase; UV: ultraviolet; VIS: visible; MN: micronucleus/micronuclei; Dox: doxorubicin; EndoIII: endonuclease III (Nth); 8-OH–G: 8-oxo–7,8-dihydroguanine; B(a)P: benzo(a)pyrene; PhIP: 2-amino-1-methyl-6-phenylimidazo[4,5-b]pyridine.

**Table 2:** Effects of plant extracts on various genetic damage endpoints, in humans, in animals (‘in vivo’), and in cultured cells (‘in vitro’).

Reference	Material tested	Analysis	Assays	System	Concentration/Dose	Result
**In Humans**						
[27]	Green tea polyphenols		Urinary 8-OH–G by HPLC	Postmenopausal women with osteoporosis	500 mg/day (capsules, 6 months)	8-OH–G ↓ over 6 months
**In Vivo**						
**Tea-Related**						
[28]	Green tea polyphenols		8-OH–G in brain by Ab assay	Rats	400 mg/day (gastric intubation, 4 weeks)	8-OH–G ↓
[29]	Green tea polyphenols	Epicatechin derivatives	CPD on skin and lymph nodes by Ab assay	Mice (NER+ and-) + UV	0.2% in drinking water (7 days before UV irradiation)	Enhanced removal of CPDs in NER-proficient mice
[30]	Green tea extract		MN in polychromatic erythrocytes	Mice + Cr(VI)	30 mg/kg (one dose—gavage)	MN ↓
[31]	Green tea polyphenols		Comet assay with Fpg on blood; 8-OH–G in brain by HPLC	Rats + acrylonitrile	0.4% in diet (1 week before acrylonitrile and then throughout acrylonitrile treatment for 28 days)	↓ Fpg-sensitive sites and 8-OH–G ↓
[32]	*Calluna vulgaris* polyphenol extract		CPDs in skin by Ab assay	Mice + UV(B)	4 mg/cm^2^ (30 min before exposure to UV, repeated on 10 days)	CPDs ↓
[33]	*Podophyllum hexandrum* extract	Total phenolics	Alkaline halo assay; DNA repair (SB rejoining—PCR assay)	Thymocytes from γ-irradiated mice	15 mg/kg (one dose, i.p.)	Protection against γ-ray-induced DNA SBs and accelerated rejoining
[34]	*Cotinus coggyria* extract		Comet assay on liver	Rats + pyrogallol	0.5–2 g/kg (single dose, i.p.)	SBs at highest dose of extract alone: protection against pyrogallol-induced SBs at 0.5 g/kg
**In Vitro**						
**Tea-Related**						
[35]	Green tea polyphenols		Comet assay	Melanoma cell lines	20–60 μg/mL (time)	40, 60 μg/mL; DNA SBs ↑
[36]	Green tea extract		Comet assay	Human laryngeal carcinoma cell line (HEp2) + drug-resistant cell line CK2	1× = 2 g/200 mL H_2_O_2_ Concentration tested = 0.1×	SBs ↑ at 72 h, not 48 h
**Lamiaceae**						
[37]	Citrus and rosemary bioflavonoid extract	Total polyphenols	Comet assay	HaCaT (human keratinocytes) + UV(B)	100 μg/mL	Pre-treatment: UV(B)-induced DNA SBs ↓
			MN	Human lymphocytes + X-ray	1 mg/mL	X-ray induced MN ↓
[38]	*Thymus vulgaris* extract		Comet assay and γ-H2AX by Ab	Human skin model exposed to UV(B)	1.8 μg/mL	Protection against DNA damage
[39]	*Thymus vulgaris* extract		Comet assay 24 h after UV	NCTC (human keratinocytes) + UV(A) or UV(B)	1.82 μg/mL	DNA SBs ↓
			MN			No effect seen
			γ-H2AX by Ab			No effect seen
[40]	Lemon balm extract	Polyphenols	Comet assay and γ-H2AX by Ab assay	Human keratinocytes + UV(B)	15–100 μg/mL	DNA SBs ↓ (100 μg/mL); γH2AX ↓ (15 μg/mL)
[41]	*Ocimum sanctum* extract (“Holy basil”)	Total phenolics	Comet assay	SH-SY5Y (human neuroblastoma) cells	75 μg/mL	H_2_O_2_-induced DNA SBs ↓
[42]	Various *Lamiaceae* leaf extracts	Total polyphenols, flavonoids	Comet assay	HepG2 (human liver carcinoma) cells + CdCl_2_	50–350 μg/mL for 4 h	Dose-dependent decrease in Cd-induced DNA SBs
**Fruits and Berries**						
[43]	Strawberry extract	Anthocyanins	Comet assay	Human dermal fibroblasts exposed to UV(A)	0.05–0.5 mg/mL	Protection against DNA SBs at 0.25, 0.5 mg/mL
[44]	Strawberry extract	Total phenolics, flavonoids, anthocyanins, vitamin C, β-carotene	Comet assay	Human dermal fibroblasts exposed to H_2_O_2_	0.5 mg/mL	DNA SBs ↓
[45]	*Vaccinium* berries extract	Total polyphenols and anthocyanins	Comet assay	A549 (human lung adenocarcinoma) cells	21–167 μg/mL	Dose-dependent protection against DNA SBs induced by t-BOOH
[46]	Blackcurrant extract		Comet assay (H_2_O_2_ resistance)	TK6 (human lymphoblastoid) cells	0.5–3 mg/mL	H_2_O_2_-induced DNA SBs ↓
			MN ± H_2_O_2_		1 mg/mL	H_2_O_2_-induced MN ↓
[47]	Various apple polyphenol s extract	Monomeric polyphenols oligosaccharides and oligomeric procyanidins.	Comet assay with Fpg	Caco2 (colon carcinoma) cells	1–100 μg/mL	Menadione-induced DNA SBs and Fpg-sensitive sites ↓ Greatest protection at low concentrations; with some extracts, damage ↑ at high doses
[48]	Polyphenol extracts of Australian fruits	Phenolic acids and anthocyanins	MN	HT29 (human colon adenocarcinoma) cells	0.5–1 mg/mL	MN ↑ with one extract
[49]	Red wine extract		Comet assay	HUVECs (human umbilical vein endothelial) cells + *t*-BOOH	25 μg/mL	DNA SBs ↓
**Honey-Related**						
[16]	Honey extract	Phenolic compounds	Comet assay with EndoIII, Fpg	Bronchial epithelial and neuronal cells	5 μg/mL	Pesticide (glyphosate, chlorpyrifos)-induced damage (SBs, EndoIII and Fpg sites) ↓
			Cellular DNA repair			Protection against inhibition of repair of DNA SBs by pesticides
[50]	Propolis extr		Comet assay	Fibroblasts	0.1–0.3 mg/mL	γ-Ray-induced DNA SBs ↓
[51]	Propolis		Comet assay + Fpg, EndoIII	Human gastric cancer cell line AGS	0.3 µg/mL	High DNA damage, suppressed by antioxidants or catalase

Ab: antibody; CPD: cyclobutane pyrimidine dimer; NER: nucleotide excision repair; i.p.: intraperitoneal; *t*-BOOH: *tert*-butyl hydroperoxide; HUVEC: human umbilical vein endothelial cell.

**Table 3:** Effects of individual polyphenolic compounds on various genetic damage endpoints, in humans, in animals (‘in vivo’), and in cultured cells (‘in vitro’).

Reference	Material Tested	Assays	System	Concentration/Dose	Result
**In Humans**					
[52]	Epigallocatechin gallate	8-OH–G in leukocyte DNA (HPLC/UV/MS)	Prostate cancer patients	800 mg/day (3 to 6 weeks before surgery)	Decrease in 8-OH–G not significant
[53]	Xanthohumol (drink)	Comet assay and urinary 8-OH–G (UPLC)	Cross over intervention trial, healthy subjects	12 mg/day for 14 days	FPG-sites ↓, H_2_O_2_-induced SBs ↓, 8-OH–G ↓
	Xanthohumol (pills)	Comet assay	Parallel intervention trial, healthy subjects		FPG-sites ↓, H_2_O_2_-induced SBs ↓
**In Vivo**					
[54]	Luteolin	Comet assay and MN on blood and bone marrow	Mice + ochratoxin A	2.5 mg/kg (one dose i.p.)	No effect
	Chlorogenic acid			10 mg/kg (one dose i.p.)	DNA SBs ↓; also MN ↓
	Caffeic acid			10 mg/kg (one dose i.p.)	DNA SBs ↓
[55]	Curcumin	Comet assay with FPG on bone marrow	Rats + etoposide	100 or 200 mg/kg/day (7 days, gavage)	Pretreatment → etoposide-induced DNA damage ↓
	Epicatechin			20 or 40 mg/kg/day (7 days, gavage)	Pretreatment → etoposide-induced oxidative DNA damage ↓ (less than with Curcumin) but not DNA SBs.
[56]	Ellagic acid	MN in polychromatic erythrocytes; alkaline unwinding	Swiss albino mice + cyclophosphamide	50/100 mg/kg/day (orally, 7 days)	Protection against MN formation and DNA SBs
[57]	Epigallocatechin gallate and theaflavin	Alkaline unwinding assay	Mouse skin + dimethylbenzanthracene	100 μg/mouse (topical application, 1 h)	Topical pretreatment → DNA SBs ↓
	Epigallocatechin gallate and theaflavin as NPs (PLGA)			5–20 μg/mouse (topical application, 1 h)	NP form has ~30-fold dose-advantage
[58]	Epigallocatechin gallate	γ-H2AX by Western blot and Ab and 8-OH–G by Ab assay	H1299 (human lung cancer cells) xenografts in mice	0.1%–0.5% in diet, 30 mg/kg/day injection	Dose-dependent ↑ in γ-H2AX and 8-OH–G
[59]	Silibinin	8-OH–G in various brain regions by ELISA	Diabetic mice	20 mg/kg/day i.p. (4 weeks)	8-OH–G ↓ in different regions of brain
[60]	Quercetin	MN in bone marrow and blood	Rats + PCBs	50 mg/kg/day for 25 days	PCB-induced MN ↓
[61]	Quercetin	Chrom abs and MN in bone marrow; Comet assay on blood	Mice + γ-irradiation	20 mg/kg/day for 5 days	Radiation-induced Chrom abs, SBs, MN ↓
	Rutin			10 mg/kg/day for 5 days	
[62]	Chrysin	Comet assay (hepatocytes and leukocytes)	Rats + methyl mercury	0.1, 1, 10 mg/kg/day for 45 days	MeHg-induced SBs ↓ at higher doses
[63]	Puerarin	8-OH–G in kidney by HPLC	Mice + CCl4	0.2 or 0.4 g/kg/day for 4 weeks	8-OH–G ↓
[64]	Quercetin	8-OH–G in kidney by HPLC	Rats + lead	10 mg/kg/day for 10 weeks	8-OH–G ↓
[65]	Myricitrin, Myricetin	MN (reticulocytes); Comet assay (liver, duodenum, stomach)	Mice	1, 1.5, 2 g/kg/day for 3 days	No increase in MN, SBs only in liver + myricetin
[66]	Quercetin	Comet assay on liver	Rats + DEN	10, 30, 100 mg/kg/day for 5 days	DEN-induced SBs ↓
[34]	Myricetin	Comet assay on liver	Rats + pyrogallol	255.5 μg/kg 2 h and 12 h before pyrogallol	SBs ↓ in liver
[67]	Quercitin	Comet assay on liver	Rats + acrylamide	10 mg/kg/day for 5 days	No effect of quercetin alone. Acrylamide-induced SBs ↓
		8-OH–G in liver by ELISA			No effect of quercetin alone. Acrylamide-induced 8-OH–G ↓
[68]	Naringin	Comet assay	Mice (hepatocytes and cardiocytes)	50, 250 or 500 mg/kg oral (one dose)	No effect
				50, 250 or 500 mg/kg oral (one dose) + Dau i.p.	DNA SBs induced by Dau ↓
[69]	Apigenin	Chrom abs and MN in bone marrow; comet assay on skin; DNA repair (removal of CPDs by Ab)	Mice + UV(B)	1.5–3 mg/cm^2^ (24 h; during UV irradiation)	Chrom abs and MN ↓; tail length ↓. Removal of dimers apparently stimulated by apigenin
**In Vitro**					
**Tea-Related**					
[70]	Chlorogenic acid	Comet assay	HaCaT (human keratinocytes) cells + UV(B)	Not stated. Probably 5–80 μM	DNA SBs ↓
[71]	Chlorogenic acid	Comet assay	K562 (human leukaemia) cells	0.5–5 mM	DNA SBs ↑
		γ-H2AX by Ab	Chinese hamster AA8 cell line and K562	0.5 mM	γ-H2AX foci ↑
[72]	Chafuroside B (tea polyphenol)	CPDs by Ab	Human keratinocytes + UV(B)	1 μM	CPDs ↓ after 24 h
[73]	Ellagic acid	Comet assay	Prostate cancer cell lines LNCaP, DU145, BPH-1	4.5–300 μM	DNA SBs ↑ at 9 μM in BPH-1, 37 μM in DU 145, 150 μM in LnCap
[74]	Epicatechin gallate	Comet assay; MN	C6 astroglial cells	0.1–1 μM	H_2_O_2_-induced DNA SBs and MN formation ↓
[58]	Epigallocatechin gallate	γ-H2AX and 8-OH–G by Ab assay	H1299 (human lung adenocarcinoma) cells	50 μM	γ-H2AX and 8-OH–G ↑
[75]	Metabolites of quercetin, chlorogenic acid	Comet assay	LT97 (human colorectal adenoma) cells + cumene hydroperoxide	2.5 μM/5 μM	Decrease in DNA SBs
[76]	Epigallocatechin gallate	Comet assay	HeLa (human cervical cancer) cells, p53R (cells with p53 reporter)	10, 20 μg/mL	DNA SBs ↑
[77]	Ethyl gallate	Comet assay	Human carcinoma cell line KB	20–50 μg/mL	DNA SBs ↑
[78]	Tannic acid	Comet assay with Fpg	Human neutrophils	10–150 μM	DNA SBs ↑ (dose-dependent); weak effect (↑) in TPA-stimulated cells. Fpg sites also ↑, but ↓ in TPA-stimulated cells
	Resveratrol				DNA damage (SBs) ↑ (dose-dependent); but ↓ (dose-dependent) in TPA-stimulated cells. Same pattern with FPG sites
[36]	Epigallocatechin gallate; Epicatechin gallate	Comet assay	HEp2 (human laryngeal carcinoma cell line)	50 μM	With either ECG or EGCG, SBs ↓ at 48 h (from background); no effect at 72 h
			CK2 (drug resistant, from HEp2)		No effect at 48 or 72 h
**Curcumin**					
[79]	Curcumin; Ellagic acid	Comet assay	HeLa (human cervical cancer) cells	25 μM	DNA SBs ↑ (with both together; not significant alone)
[80];	Curcumin	Chrom abs and PCC	Human lymphocytes, with/without stimulation	0.14–7 μM	Radioprotective effects seen for both reagents in PCC assay (non-cycling cells) Radiosensitisation of cycling cells (chrom abs) by both reagents
	Resveratrol			2.2–220 μM	
[81]	Curcumin	8-OH–G by Ab assay	Smooth muscle cells	up to 10 μM	8-OH–G ↑
[82]	Quercetin; Curcumin	γ-H2AX by Ab assay	HT1080 human fibrosarcoma cell line	30 and 80 μM Quercetin; 10 and 15 μM Curcumin,	Significant increases in γ H2AX
		MN		30 μM Quercetin; 10 μM Curcumin	Significant increases in MN. (Quercetin less effective.)
[83]	Soy isoflavones	γ-H2AX by Ab assay	LNCaP (human prostate cancer) cells	10 μg/mL	No effect on H2AX
	Curcumin			25 μg/mL	γ-H2AX ↑
[84]	Polyphenols	Comet assay	Lymphocytes + B(a)P	5 μg/mL	DNA SBs ↓
	Curcumin			5 and 10 μg/mL	DNA SBs ↓
[85]	Curcumin	Comet assay	HCT-116 (human colon cancer) cells	50 μM	DNA SBs ↑
[86]	Curcumin	Comet assay	K562 (human leukaemia) cells	12.5–200 μM	DNA SBs ↑
**Resveratrol**					
[87]	Resveratrol	Chrom abs	Human lymphocytes + aflatoxin	10–100 μM	No effect of resveratrol alone. Dose-dependent decrease in aflatoxin-induced chrom abs
[88]	Resveratrol	MN; Comet assay	Human bronchial epithelial cell line HBE + Na arsenite	5 μM	↓ DNA SBs and MN induced by arsenite
[89]	Resveratrol	γ -H2AX by Ab assay	HCT-116 (human colon cancer) cells	25 μM	γ-H2AX foci ↑: DNA damage due to toposiomerase II poisoning
[90]	Resveratrol	γ -H2AX by Ab assay	Prostate epithelial cells	5 μM	Ionising radiation-induced damage enhanced
[91]	Resveratrol	Comet assay	Rat astrocytes + ethanol	1–10 μM	↓ DNA SBs induced by ethanol
**Lamiaceae**					
[39]	Thymol	Comet assay 24 h after UV	NCTC (human keratinocytes) + UV(A) or UV(B)	1 μg/mL	DNA SBs ↓
		MN			No effect seen
		γ-H2AX by Ab assay			No effect seen
**Flavonoids**					
[92]	Naringin	Chromosome aberrations	Human lymphocytes treated with Cd	1, 2 μg/mL	Cd-induced chrom abs ↓
		SCE			No significant effect on SCE
[93]	Rutin	Comet assay	Rat hepatic cell line HTC	10–810 μg/mL (24 h)	SBs at highest concentration
		MN			No significant increase in MN—but protection against MN induced by B(a)P
[94]	Quercetin; Rutin	ã-H2AX by Ab assay	V79 lung fibroblast hamster cells	100 μg/mL for 12 h	Massive foci, results of lethality
[95]	Kaempferol	Comet assay	HL-60 human leukemia cells	75 μM, 6–48 h	SBs induced
[96]	Quercetin	Comet assay	Lymphocytes from healthy subjects and colon cancer patients, + food mutagens PhIP and IQ	100, 250, 500 μM	SBs induced by PhIP or IQ ↓
	Rutin			50, 250, 500 μM	
[97]	Fisetin, Kaempferol; Galangin; Quercetin; Luteolin; Chrysin; 7-hydroxyflavone; 7,8-dihydroxyflavone; Baicalein; Rutin	Comet assay; MN	HepG2 (human liver carcinoma) cells + B(a)P	2.5–25 μM	SBs induced by B(a)P ↓ (all except rutin); MN induced by B(a)P ↓ (all except rutin); Fi>Qu>Ga>Ka>Lu (more effective group); Ch, 7Fl, 7,8Fl, Ba (less effective group)
[98]	Fisetin	Comet assay	Human hepatic Huh-7 cells	60 μM	SBs ↑
[99]	Kaempferol	Comet assay	Human osteosarcoma cells U2-OS	50, 100, 150 μM	SBs ↑ (not quantitated)
[65]	Myricitrin	MN	TK6 (human lymphoblastoid) cells	20–500 μg/mL for 24 h	MN ↑ (Dose-dependent)
	Myricetin			2.5–75 μg/mL for 24 h	MN ↑ (significant?)
[100]	Quercetin and rutin	Comet assay	Human hepatoma cell line HepG2	0.1, 1 and 5 μg/mL (2 h of treatment)	No induction of SBs (quercetin and rutin alone)
			HepG2 + Aflatoxin B, MMS, Dox	Pre-, co- and post-treatment	DNA damage induced by AFB1, MMS, Dox ↓ in all treatment conditions
[101]	Quercetin	Comet assay, 8-OH–G (HPLC)	Human hepatoma cell line HepG2 cells	0.1, 1 and 5 μg/mL (24 h of treatment)	No effect
			HepG2 cells + HgCl_2_ and MeHg	Pre-, co- and post-treatment	DNA damage induced by HgCl_2_ and MeHg ↓ in pre- and co-treatment
[102]	Quercitrin	Comet assay	Mouse epidermal cell line JB6 + UV(B)	10, 20 and 80 μM, 30 min	No effect
				10, 20 and 80 μM, 30 min + UV(B)	UV(B)-induced SBs ↓
[51]	Galangin, chrysin	Comet assay + FPG, EndoIII	AGS human gastric adenocarcinoma cells	20 μM (1 h)	Base oxidation ↑
[69]	Apigenin	Comet assay: Chrom abs; MN	HaCaT human keratinocytes + UV(B)	15–25 μg/mL	DNA damage ↓, Chrom abs ↓, MN ↓

PCB: polychlorinated biphenyls; chrom ab: chromosome aberration; DEN: diethylnitrosamine; Dau: Daunorubicin; TPA: tetradecanoyl phorbol acetate; ECG: epicatechin gallate; EGCG: epigallocatechin gallate; PCC: premature chromosome condensation; SCE: sister chromatid exchange; IQ: 2-amino-3-methylimidazo[4,5-f]quinolone; MMS: methylmethanesulphonate; AFB1: aflatoxin B1.

## Abbreviations

SB	strand break
Fpg	formamidopyrimidine DNA glycosylase
EndoIII	endonuclease III (Nth)
8-OH–Gua (8-OH–G)	8-oxo–7,8-dihydroguanine
PBMN	peripheral blood mononuclear
NER	nucleotide excision repair
Ab	antibody
NP	nanoparticle
Dox	doxorubicin
B(a)P	benzo(a)phenol
CPD	cyclobutane pyrimidine dimer
*t*-BOOH	*tert*-butyl hydroperoxide
PCB	polychlorinated biphenyls
DEN	diethylnitrosamine
TPA	tetradecanoyl-phorbol acetate
